# Hepatic Doppler Perfusion Index in Healthy Adults: Standardization, Physiological Reference Limit, and Clinical Perspectives

**DOI:** 10.3390/diagnostics16121840

**Published:** 2026-06-14

**Authors:** Christian Lueders, Johannes Gladitz, Nils Eckstein, Judith Schulz, Thomas Kiefer, Heinz Völler, Carsten-Heinrich Weylandt, Daniel Merkel

**Affiliations:** 1 Klinik am See, Rehabilitation Center for Internal Medicine, Seebad 84, 15562 Rüdersdorf bei Berlin, Germany; nils.eckstein@klinikamsee.com (N.E.); thomas.kiefer-trendelenburg@klinikamsee.com (T.K.); heinz.voeller@klinikamsee.com (H.V.); 2 Department of Gastroenterology, Brandenburg Medical University Theodor Fontane, 16816 Neuruppin, Germany; karsten.weylandt@mhb-fontane.de (C.-H.W.); daniel.merkel@mhb-fontane.de (D.M.); 3 Brandenburg Institute for Clinical Ultrasound (BICUS), Brandenburg Medical Universitiy Theodor Fontane, 16816 Neuruppin, Germany; 4 Statistical Consulting Dr. Gladitz, 10119 Berlin, Germany; 5 Department of Rehabilitation Medicine, Faculty of Health Sciences Brandenburg, University of Potsdam, 14469 Potsdam, Germany; 6 Department of Internal Medicine, Caritas-Klinik Dominikus, 13467 Berlin, Germany

**Keywords:** Doppler perfusion index, hepatic perfusion, Doppler ultrasonography, hepatic artery, portal vein, reference values, standardization

## Abstract

**Background/Objectives:** The Doppler perfusion index (DPI) quantifies the ratio of arterial to total hepatic blood flow and reflects hepatic hemodynamic balance. Its clinical applicability is limited by insufficient standardization and the absence of clearly defined physiological reference conditions. This study aimed to establish an upper physiological reference limit for the DPI and to assess its dependence on standardized physiological conditions in healthy adults. **Methods:** In this prospective study, 44 healthy adults underwent Doppler ultrasonography under standardized conditions (fasting/resting, post-exercise, postprandial). Volumetric blood flow was measured in the portal vein and via the proper hepatic artery and, where feasible, the common hepatic artery. The DPI was calculated as the ratio of arterial to total hepatic inflow. Nonparametric statistical methods were applied. **Results:** After exclusion of participants with non-standard hepatic arterial anatomy, 39 individuals were analyzed. The DPI varied across physiological conditions, reflecting changes in the relative contributions of arterial and portal venous inflow. Under fasting/resting conditions, values based on the proper hepatic artery showed low variability (mean 0.242 ± 0.057) and normal distribution (Shapiro–Wilk *p* = 0.625). The empirically derived 90th percentile was 0.30. Measurements based on the common hepatic artery were higher and more variable. **Conclusions:** The DPI is a physiologically dynamic parameter whose clinical use requires standardized measurement conditions. Under defined protocols, a value of approximately 0.30 may be considered an upper physiological reference limit. Standardization of acquisition and use of the proper hepatic artery enable reproducible and interpretable measurements. This provides a methodological basis for further clinical applications, including oncological contexts in which functional alterations of hepatic perfusion may be relevant.

## 1. Introduction

The liver is characterized by a unique dual blood supply consisting of portal venous and arterial inflow. The balance between these inflow pathways is regulated by the hepatic arterial buffer response, which maintains relative constancy of total hepatic blood flow despite fluctuations in portal venous flow (Eipel et al., 2010; Lautt, 2009) [1,2]. Disruption of this balance represents a key feature of numerous liver diseases and reflects functionally relevant alterations in hepatic perfusion.

The Doppler perfusion index (DPI) provides a noninvasive quantitative measure of the ratio between arterial and total hepatic blood flow. In various clinical contexts, the DPI has been proposed as a potential marker for malignant hepatic infiltration, inflammatory liver disease, and fibrotic remodeling of the liver (Iliopoulos et al., 2008; Kakkos et al., 2000; Leen, 1999; Riahinezhad et al., 2021) [3,4,5,6]. In particular, early studies suggested that increased DPI values may reflect hemodynamic alterations associated with hepatic tumor involvement below the spatial resolution of conventional morphological imaging (Leen, 1999; Leen et al., 2000) [5,7]. However, the available literature is methodologically heterogeneous and reports inconsistent results, precluding a definitive assessment of the clinical utility of the DPI (Kopljar et al., 2014; Roumen et al., 2005) [8,9].

A major limitation to clinical application is the absence of reliable reference values derived from healthy individuals. Published measurement protocols frequently lack precise specification of physiological conditions. Although examinations are commonly described as being performed under fasting and resting conditions, the extent to which the DPI itself varies across different physiological states remains largely unknown. This limitation is of particular relevance because the DPI represents a ratio of two volumetric flow measurements and therefore depends on the combined physiological variability of both arterial and portal venous inflow (Curran-Everett, 2013; Goldstein, 1999) [10,11].

An additional methodological uncertainty concerns the optimal arterial measurement site. In the literature, the arterial component of the DPI has been derived from either the common hepatic artery or the proper hepatic artery, often without explicit reporting of the measurement location. Moreover, anatomical variability of the hepatic arterial supply represents a further confounding factor that may differentially affect measurements based on these vessels. Variants of the hepatic arterial anatomy have been reported in at least 20% of individuals, while large imaging series have demonstrated somewhat higher prevalences (Choi et al., 2021; Hiatt et al., 1994; Michels, 1966; Varotti et al., 2004) [12,13,14,15].

Against this background, the present study was designed to address these methodological limitations. The primary aim was to define an upper physiological reference limit of the DPI in healthy adults under standardized baseline conditions. In addition, the study aimed to quantify the dependence of the DPI on defined physiological states and to evaluate the methodological implications of different arterial measurement sites. By providing a standardized physiological reference framework, the study seeks to support reproducible and clinically interpretable application of the DPI in hepatic Doppler ultrasound and to facilitate further translational and clinical evaluation.

## 2. Materials and Methods

### 2.1. Investigator and Equipment

All examinations were performed by a single investigator with certification in abdominal ultrasonography according to the guidelines of the German Society for Ultrasound in Medicine (DEGUM).

Doppler ultrasound examinations were performed using a Canon Aplio i300 system (Canon Medical Systems, Tokyo, Japan) with a 1–5 MHz convex transducer. Physical exercise was performed on a clinical bicycle ergometer (ergoselect 200, ergoline GmbH, Bitz, Germany) with continuous acquisition of workload and cadence. Cardiovascular monitoring was performed using a spiroergometry system (CORTEX Biophysik GmbH, Leipzig, Germany), including 12-lead electrocardiography, heart rate, noninvasive blood pressure, and pulse oximetry.

### 2.2. Study Design

This prospective, standardized, intraindividual observational study aimed to assess hepatic blood supply and to derive the Doppler perfusion index (DPI) using Doppler ultrasound-based volumetric flow measurements under defined physiological conditions in healthy volunteers.

All participants underwent a standardized examination protocol comprising three consecutive physiological states (fasting/resting, post-exercise, and postprandial). All measurements were performed on the same study day and followed a predefined sequence.

The study followed a repeated-measures design, with each participant serving as their own control under non-interventional physiological conditions.

### 2.3. Participants

The study was approved by the Ethics Committee of the Brandenburg Medical School Theodor Fontane (reference number 208052024-BO-E). All participants were fully informed about the objectives and procedures of the study prior to enrollment and provided written informed consent. The study was registered in the German Clinical Trials Register (DRKS; registration number DRKS00039463).

Healthy adult volunteers were prospectively recruited. Eligibility criteria included an unremarkable general and nutritional status and absence of relevant medical conditions.

Exclusion criteria:

- known or reported liver disease,
- cardiovascular comorbidities,
- relevant metabolic or systemic diseases,
- acute illness at the time of examination,
- a history of abdominal surgery with potential impact on hepatosplanchnic hemodynamics.

Permitted prior abdominal procedures were limited to appendectomy and surgical interventions in the genital or urogenital region.

At the time of examination, none of the participants were taking regular medication except for hormonal contraceptives.

Before study initiation, all participants underwent a baseline clinical assessment including physical examination, resting electrocardiography, blood pressure measurement, and screening abdominal ultrasound.

### 2.4. Study Conditions and Examination Protocol

All participants underwent a standardized examination protocol comprising three predefined physiological conditions. All examinations were performed on the same study day and followed a predefined temporal sequence.

#### 2.4.1. Fasting/Resting Condition

The first examination was performed under fasting and resting conditions after a minimum fasting period of eight hours. Participants were examined in the supine position after resting in this position for at least 30 min. Doppler ultrasound measurements were obtained during quiet breathing with brief breath-hold at end expiration.

#### 2.4.2. Standardized Physical Exercise

Following completion of the fasting measurements, participants underwent standardized physical exercise on a clinical bicycle ergometer. Exercise was performed as aerobic endurance exercise within the basic endurance range, consisting of a ten-minute warm-up followed by a steady-state phase.

Exercise intensity was prospectively standardized using a subjective exertion criterion based on the “talk test” (Reed & Pipe, 2014) [16]. Participants were instructed to regulate their exercise intensity such that relaxed conversation remained possible throughout the exercise period, corresponding to moderate aerobic intensity. For orienting estimation of individual exercise intensity, a target heart rate was calculated prior to exercise using the Karvonen formula (Karvonen et al., 1957) [17], with maximal heart rate estimated in an age- and sex-specific manner according to Nes et al. (Nes et al., 2013) [18]. Exercise was not strictly heart rate-controlled and was primarily guided by subjective perceived exertion.

Throughout the exercise period, continuous monitoring of workload, pedal cadence (rpm), and cardiovascular status was performed, including 12-lead electrocardiography, heart rate monitoring, noninvasive blood pressure measurement, and pulse oximetry. Exercise testing was conducted in a clinical setting with continuous medical supervision.

Doppler ultrasound assessment of the visceral vessels during ongoing physical exercise is technically not feasible and not comparable to stress echocardiographic procedures. Therefore, exercise was terminated abruptly, and participants were immediately placed in the supine position. Doppler ultrasound measurements were initiated without delay and performed during quiet breathing with brief breath-hold at end expiration. The entire examination was completed within a maximum of 60 s to minimize the influence of early hemodynamic recovery processes.

#### 2.4.3. Postprandial Condition

After a recovery period of at least 60 min following physical exercise, participants consumed a standardized meal under resting conditions. Meal composition was developed in consultation with a registered dietitian and prepared by a professional kitchen; vegetarian and non-vegetarian options were available. Caloric content was adjusted to body weight (approximately 10–12 kcal/kg) to ensure a standardized postprandial stimulus (Energy and Protein Requirements. Report of a Joint FAO/WHO/UNU Expert Consultation, 1985) [19].

Postprandial Doppler ultrasound examination was performed 60 min after completion of meal intake, again in the supine position and during quiet breathing with brief breath-hold at end expiration.

### 2.5. Doppler Ultrasound Methodology

#### 2.5.1. Role and Assessment of Arterial Vascular Variants

Arterial vascular variants of the liver are relevant for quantification of hepatic arterial inflow and calculation of the Doppler perfusion index (DPI), as they may affect both arterial flow distribution and assignment of Doppler measurements to specific vascular segments. Accordingly, the study protocol included a systematic assessment of arterial vascular variants under resting conditions.

Classification of hepatic arterial anatomy was performed according to the Varotti system (Varotti et al., 2004) [15]. In contrast to the Michels and Hiatt classifications (Hiatt et al., 1994; Michels, 1966) [13,14], the Varotti system differentiates right- and left-sided variants and distinguishes accessory (“a”) from replaced (“r”) arterial supply, corresponding well to practical sonographic assessment.

In the predominantly normal-weight study population, sonographic assessment of arterial variants was generally technically feasible, including variants affecting the right hepatic lobe.

**Figure 1:**
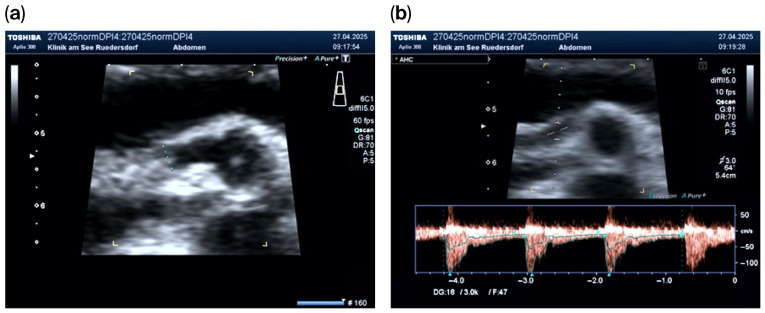
Variants of the arterial blood supply to the liver. (**a**) Monovascular hepatic arterial supply originating from the superior mesenteric artery (SMA), corresponding to Varotti type 5. (**b**) Doppler spectral profile illustrating arterial flow characteristics. Note. For classification as Varotti type 5, accessory arterial supply to the right or left hepatic lobe must be excluded. In cases with Varotti type 5 anatomy, the corresponding hemodynamic measurements were included in the quantitative analysis.

Arterial vascular variants were generally excluded from further quantitative analysis. An exception was made for monovascular variants (Varotti type 5), in which a single, clearly defined arterial inflow could be identified, allowing consistent calculation of the arterial component of the DPI (Figure 1).

#### 2.5.2. Doppler Ultrasound Assessment of the Hepatic Arteries

The Doppler ultrasound protocol for quantification of hepatic inflow, including arterial (CHA, PHA) and portal venous (PV) components, was based on a previously published and validated standard protocol for assessment of the Doppler perfusion index (DPI) and was selectively extended to address anatomical and practical limitations encountered in the study population (Lueders et al., 2024) [20].

Whenever technically feasible, the CHA was assessed in a sufficiently long, straight vessel segment distal to its origin from the celiac trunk. Measurements performed immediately at the vessel origin were avoided, as the Doppler spectrum in this region is frequently influenced by the flow profile of the celiac trunk.

**Figure 2:**
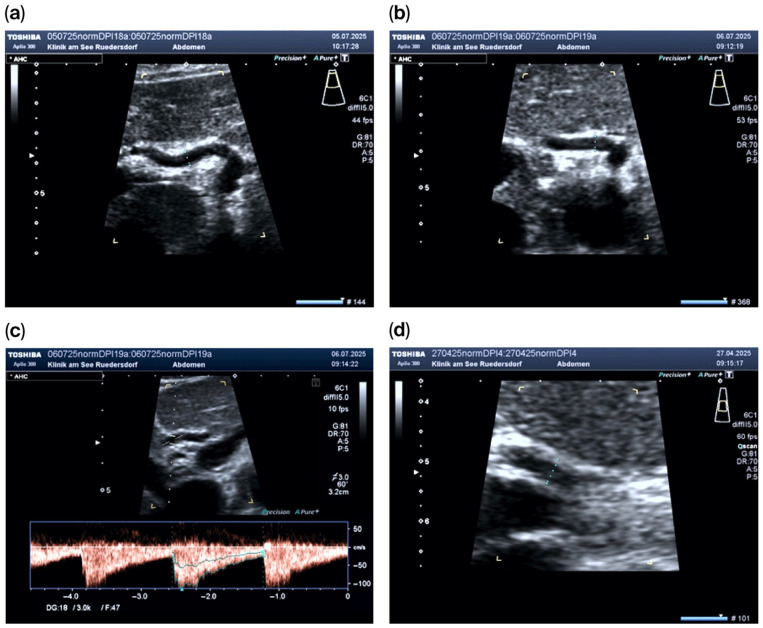
Doppler sonographic approaches to the common and proper hepatic artery. (**a**) Sinuous course of the common hepatic artery. (**b**) Transverse course of the common hepatic artery. (**c**) Doppler assessment of the proper hepatic artery. (**d**) Determination of the proper hepatic artery diameter. Note. A favorable vessel course of the common hepatic artery allows a feasible insonation angle (**a**), whereas a transverse course precludes an insonation angle ≤ 60° despite adequate B-mode visualization (**b**). The proper hepatic artery is assessed via a transverse upper abdominal approach (**c**). Due to its small diameter (typically <4 mm), accurate measurement requires high-resolution magnified imaging and application of the leading-edge method (**d**).

In this predominantly normal-weight study population, the CHA frequently exhibited a near-transverse course relative to the ultrasound beam, precluding establishment of a valid Doppler insonation angle of ≤60° despite excellent B-mode visualization (Figure 2). As Doppler measurements obtained at insonation angles > 60° are associated with substantial inaccuracy, such measurements were not considered valid. Consequently, quantitative CHA assessment was feasible only in a subset of participants, reflecting an anatomy-specific limitation of Doppler ultrasound assessment of the CHA.

Due to the small vessel diameter of the proper hepatic artery (PHA), which typically measures less than 4 mm, reliable diameter determination requires the use of high-resolution zoomed imaging and strict application of the leading-edge method (Figure 2) (Lueders et al., 2024) [20].

#### 2.5.3. Doppler Ultrasound Assessment of the Portal Vein (PV)

Doppler ultrasound assessment of the portal vein (PV) is an established component of routine clinical diagnostics and was feasible in all participants without technical limitations in the present study.

**Figure 3:**
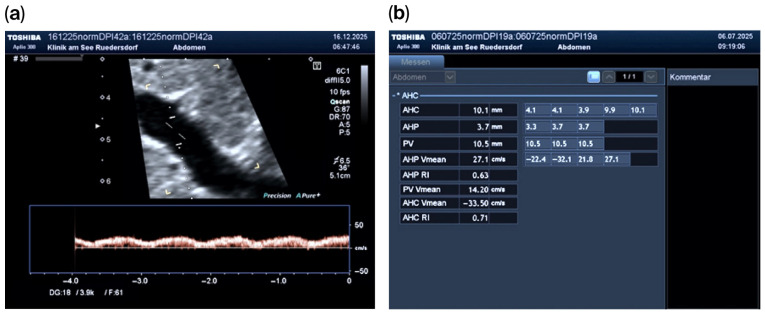
Doppler sonographic approach to the portal vein. (**a**) Doppler spectral waveform of the portal vein. (**b**) Documentation of measurement results using the standardized ultrasound preset. Note. Measurements were performed using an intercostal approach. Values of 9.9 mm and 10.1 mm represent portal vein diameter measurements.

Portal venous flow parameters were acquired exclusively using an intercostal approach, with assessment of the portal vein at its entry into the hepatic parenchyma. Doppler measurements were performed during end-expiratory breath-hold over three consecutive cardiac cycles (Figure 3a).

All measurement data were recorded using a device preset specifically programmed for this study (Figure 3b).

### 2.6. Recorded Variables and Calculation of Blood Flow and the Doppler Perfusion Index

During Doppler ultrasound examination, the following parameters were recorded in a standardized manner:

- vessel diameter (*d*, mm);
- angle-corrected Doppler flow velocity (V, cm/s);
- resistive index of the common hepatic artery (CHA) and the proper hepatic artery (PHA) (RI, dimensionless).

Doppler flow velocities were obtained using angle correction; the insonation angle itself was not documented as an independent variable.

Volumetric blood flow (*Q*) was calculated for each vessel based on the measured mean flow velocity (v_mean) and the vessel cross-sectional area (A). Cross-sectional area was derived from the measured vessel diameter assuming a circular lumen. Volumetric flow was calculated according to the following relationship:

$$Q=v_{\mathrm{mean}}\times A=v_{\mathrm{mean}}\times \pi \times {\left(\frac{d}{2}\right)}^{2}$$

For the arterial component, volumetric blood flow of either the common hepatic artery (CHA) or the proper hepatic artery (PHA) was used, depending on individual feasibility of measurement and the underlying arterial anatomy. Portal venous volumetric flow was calculated from measurements of the portal vein (PV).

The Doppler perfusion index (DPI) was defined as a dimensionless ratio of arterial inflow to total hepatic inflow. Arterial inflow corresponded to the measured volumetric flow of the CHA or, alternatively, the PHA, whereas total hepatic inflow was calculated as the sum of arterial and portal venous volumetric flow, according to the following equation:

(1) $$\mathrm{DPI}=\frac{Q_{\mathrm{arterial}}}{Q_{\mathrm{arterial}}+Q_{\mathrm{portal}\;\mathrm{venous}}}$$

The resistive index (RI) was calculated from the Doppler spectrum as a dimensionless parameter derived from the peak systolic flow velocity ($v_{\mathrm{sys}}$) and the end-diastolic flow velocity ($v_{\mathrm{dia}}$).

(2) $$RI=\frac{v_{\mathrm{sys}}-v_{\mathrm{dia}}}{v_{\mathrm{sys}}}$$

All calculations were based exclusively on measurements obtained under the predefined standardized examination conditions. The arterial measurement site used in each case (CHA or PHA) was documented and considered in subsequent analyses.

### 2.7. Statistics

The IBM SPSS 29 statistics package was used for statistical analysis. Descriptive parameters such as arithmetic mean, standard deviation, median, minimum and maximum, interquartile range, and coefficient of variation were used as parameters to characterize statistical distributions for metric measurements, and percentages were used for categorical variables. Empirically calculated percentiles were used to determine the upper limits of the normal ranges (90th percentiles). In addition, 10,000 bootstrap simulations were used to quantify the uncertainty of the calculated percentiles.

The Shapiro–Wilk test was used to test the statistical distributions of the DPIs for the two arteries (CHA and PHA) under the three physiological conditions for the presence of a normal distribution. Boxplots were used for graphical illustration.

**Figure 4:**
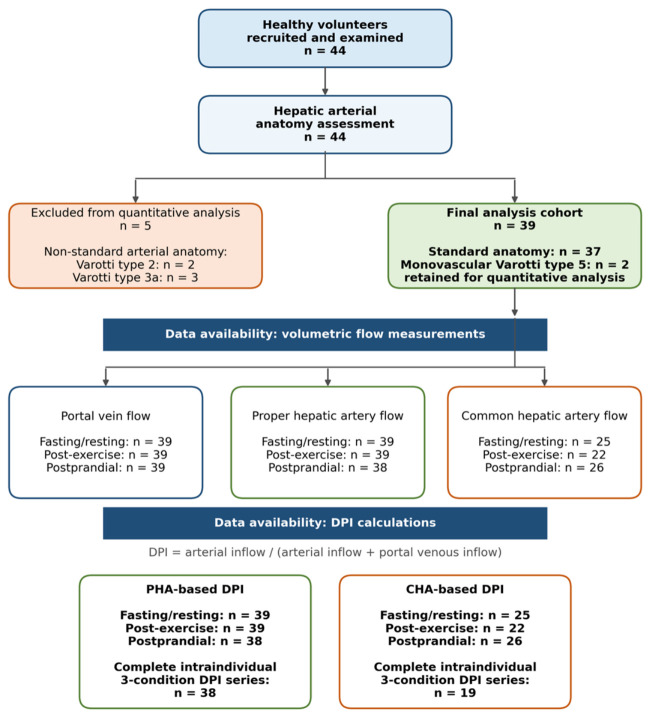
Study Flow and Data Availability. Flow diagram illustrating participant recruitment, assessment of hepatic arterial anatomy, exclusion of participants with non-standard arterial variants, final analysis cohort, and availability of volumetric flow and Doppler perfusion index (DPI) datasets for the portal vein (PV), proper hepatic artery (PHA), and common hepatic artery (CHA) under fasting/resting, post-exercise, and postprandial conditions. Complete intraindividual 3-condition DPI series are indicated for both PHA-based and CHA-based DPI analyses.

Participant recruitment, assessment of hepatic arterial anatomy, exclusion of participants with non-standard arterial variants, and availability of datasets for volumetric flow and DPI analyses are summarized in Figure 4.

## 3. Results

### 3.1. Study Population and Arterial Vascular Variants

A total of 44 healthy adults were enrolled. Arterial variants were identified in 7/44 participants during resting Doppler assessment.

According to the Varotti classification, variants included two type 2, three type 3a, and two type 5 cases. In the type 2 variant, differentiation between accessory and replaced anatomy was not feasible.

Participants with non-standard hepatic arterial anatomy were excluded, except for two cases with origin of the common hepatic artery from the superior mesenteric artery (Type 5). Consequently, 39 participants were included in subsequent analyses.

### 3.2. Study Population and Baseline Characteristics

After exclusion of participants with non-standard hepatic arterial anatomy, 39 healthy adults were included in the final analysis. Of these, 21 were male (53.8%) and 18 female (46.2%). Mean age of the study population was 37.7 ± 10.5 years (range 18–58 years).

Baseline characteristics of the study population are summarized in Table 1.

Mean body height was 174.1 ± 9.7 cm, and mean body weight was 69.0 ± 12.8 kg, corresponding to a mean body mass index of 22.7 ± 3.3 kg/m^2^. Mean body surface area calculated according to the DuBois formula was 1.82 ± 0.20 m^2^. Overall, anthropometric parameters indicated a predominantly normal-weight study population without relevant extreme values.

Sex-specific analysis showed a mean age of 35.4 ± 11.4 years in male participants and 40.4 ± 8.8 years in female participants. Men exhibited greater mean body height (180.0 ± 8.5 cm) and body weight (75.8 ± 10.5 kg) compared with women (167.1 ± 5.6 cm and 61.1 ± 10.6 kg, respectively), whereas body mass index differed only marginally between sexes.

### 3.3. Exercise and Performance Parameters

Standardized physical exercise was completed by all 39 participants without adverse events. At rest, mean systolic blood pressure was 110.3 ± 11.3 mmHg and mean diastolic blood pressure was 77.8 ± 8.3 mmHg. During exercise, systolic blood pressure increased as expected to 156.5 ± 20.0 mmHg, whereas diastolic blood pressure remained largely unchanged at 75.6 ± 13.8 mmHg.

Mean absolute workload during exercise was 142.6 ± 50.3 W (range, 75–250 W). When normalized to body weight, mean workload was 2.04 ± 0.54 W/kg, corresponding to a moderate aerobic endurance workload within the predefined moderate aerobic endurance range.

Mean target heart rate calculated using the Karvonen formula was 152.4 ± 6.4 beats/min. The heart rate achieved during exercise was 144.1 ± 9.3 beats/min, corresponding on average to slightly below the calculated target value. Individual achieved heart rates ranged from 122.4 to 164.8 beats/min, whereas calculated target values ranged from 140.8 to 165.1 beats/min.

Exercise intensity was not strictly controlled based on target heart rate but was primarily guided by subjective criteria consistent with a moderate aerobic workload. Detailed cardiovascular and exercise performance parameters are provided in Supplementary Tables S1 and S2.

### 3.4. Vessel Diameters and Resistive Indices

Vessel diameters of the common hepatic artery (CHA), proper hepatic artery (PHA), and portal vein (PV) were measured under all three physiological conditions (rest, post-exercise, postprandial). In addition, resistive indices (RI) of the CHA and PHA were recorded; no RI was determined for the portal vein.

Measured vessel diameters showed low variability across all vessels and remained within physiological ranges. Condition-dependent changes in vessel caliber were observed without evidence of extreme outliers or technically related measurement artifacts. As expected, the PHA consistently exhibited smaller diameters than the CHA, whereas the portal vein showed the largest calibers.

Resistive indices of the CHA and PHA remained within established normal ranges across all physiological conditions. Condition-dependent modulation of RI values was observed without indication of pathological resistance profiles.

Detailed results for vessel diameters and resistive indices, stratified by vessel and physiological condition, are provided in the Supplementary Material (Supplementary Tables S3 and S4).

### 3.5. Hepatic Volumetric Blood Flow Under Different Physiological Conditions

Hepatic volumetric blood flows were calculated from angle-corrected Doppler flow velocities (time-averaged mean velocity, TAMV) and measured vessel diameters as described in Section 2.6. Quantitative assessment included portal venous inflow via the portal vein (PV) and arterial inflow via the proper hepatic artery (PHA) and—where technically valid—the common hepatic artery (CHA).

PV and PHA volumetric flow measurements were successfully obtained in all participants included in the hemodynamic analysis (*n* = 39). In contrast, valid CHA-based volumetric flow assessment was feasible in only 19 participants; CHA-derived volumetric flow data are therefore available exclusively for this subgroup.

For analyses requiring complete datasets across all three physiological conditions, only these 19 participants could be included. However, valid CHA measurements were available in additional participants under individual physiological conditions. Consequently, the number of observations reported for condition-specific analyses differs from the number of participants available for complete repeated-measures analyses.

Under fasting resting conditions, the arterial contribution to total hepatic inflow calculated on the basis of the PHA averaged 24.4 ± 5.6%. PHA-based arterial volumetric flows under these conditions showed a relatively narrow distribution without extreme individual values, whereas CHA-based volumetric flows demonstrated greater variability.

Following standardized physical exercise, both portal venous and arterial inflow decreased concordantly compared with resting conditions. This pattern was observed in both PHA- and PV-based measurements.

In the postprandial state, portal venous volumetric flow increased relative to resting conditions, whereas arterial inflow decreased. This reciprocal behavior of portal venous and arterial inflow is consistent with the hepatic arterial buffer response and was observed throughout the study population.

**Figure 5:**
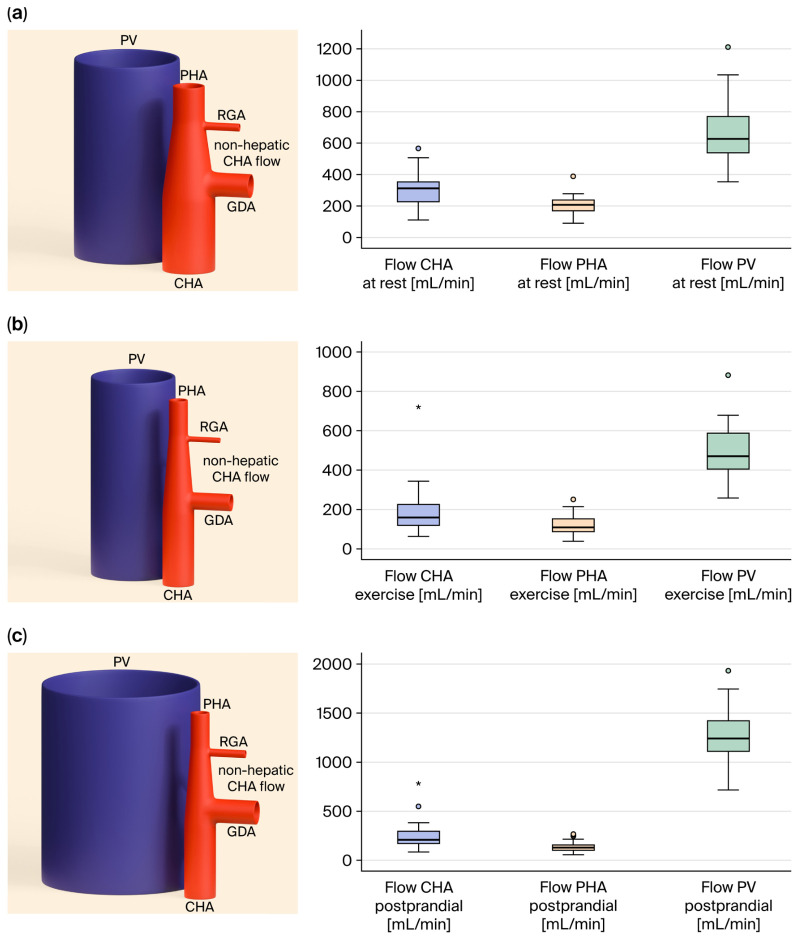
Volumetric blood flow in hepatic inflow vessels under different physiological conditions. Volumetric blood flow in the common hepatic artery (CHA), proper hepatic artery (PHA), and portal vein (PV). (**a**) Rest. (**b**) Exercise. (**c**) Postprandial state. Note. Boxplots depict median, interquartile range, and outliers. Cirlces indicate outliers and stars indicate extreme outliers. For each condition, flows of all vessels are displayed on a common *y*-axis; due to the predominance of portal venous flow, differences between CHA and PHA are visually attenuated. Adjacent vessel schematics provide a qualitative visualization of relative flow using vessel diameter as a surrogate and are not drawn to scale.

Complete results of calculated volumetric blood flows, stratified by vessel (PV, PHA, CHA) and physiological condition (rest, post-exercise, postprandial), are presented in Supplementary Table S5. Underlying Doppler flow velocities are provided in Supplementary Table S6. A graphical overview of condition-dependent changes in hepatic inflow is shown in Figure 5.

### 3.6. Doppler Perfusion Index Under Different Physiological Conditions

The Doppler perfusion index (DPI) was calculated as the ratio of arterial to total hepatic inflow using PHA- and, where available, CHA-based measurements. Numerical values are summarized in Table 2.

Under fasting resting conditions, the PHA-based DPI showed a narrow distribution with a mean value of 0.242 ± 0.057. DPI values were largely confined to a narrow interquartile range without pronounced extreme values.

Following standardized physical exercise, DPI decreased compared with resting conditions for both PHA- and CHA-based calculations. This change was overall moderate and occurred in the context of a concordant reduction in both arterial and portal venous volumetric blood flow.

In the postprandial state, a greater change in DPI was observed compared with post-exercise conditions. DPI values were shifted toward lower ranges overall, indicating a clear dependence of DPI on the examination condition. This stronger condition dependence resulted from the reciprocal changes in the underlying volumetric flows, with an increase in portal venous inflow and a simultaneous reduction in arterial inflow, consistent with the hepatic arterial buffer response.

In addition to the location of the distributions, dispersion of DPI values was assessed descriptively using robust measures of variability. Across all physiological conditions, the PHA-based DPI exhibited a smaller interquartile range and lower relative variability compared with the CHA-based DPI. In contrast, CHA-based DPI values consistently showed wider interquartile ranges and greater relative dispersion. This pattern was observed under resting conditions, following physical exercise, and in the postprandial state.

**Figure 6:**
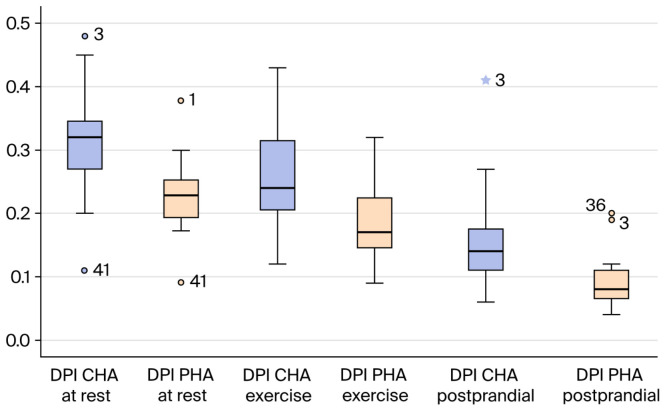
Doppler perfusion index under different physiological conditions. Distributions of the Doppler perfusion index (DPI) calculated from the common hepatic artery (CHA) and the proper hepatic artery (PHA). DPI values are shown for the fasting/resting state, post-exercise, and postprandial state. Note. Boxplots depict median, interquartile range, and outliers. CHA-based DPI values are higher and show greater dispersion than PHA-based values across all conditions. The number of observations differs between measurement sites (PHA, *n* = 38; CHA, *n* = 19 complete datasets) due to limited feasibility of valid CHA volumetric flow assessment. Circles indicate outliers and stars indicate extreme outliers; adjacent numbers represent case labels generated by SPSS.

Numerical results of DPI calculations are summarized in Table 2. Condition- and measurement site-dependent distributions of DPI values are illustrated in Figure 6.

### 3.7. Reference Analysis and Definition of the Upper Physiological Reference Limit of the DPI

The aim of the reference analysis was to define an upper physiological reference limit of the Doppler perfusion index (DPI) in healthy adults under standardized examination conditions. The fasting/resting state was defined as the reference condition, as it represents the basal physiological state without acute exercise- or meal-induced modulation of splanchnic hemodynamics.

For the reference analysis, only PHA-based DPI values were considered. The proper hepatic artery represents the direct arterial inflow to the hepatic parenchyma distal to extrahepatic branches and therefore reflects the liver-specific arterial contribution to total hepatic inflow. In addition, complete PHA-based measurements were available for all participants included in the reference analysis. CHA-based DPI values were not used for reference definition due to incomplete data availability and greater variability.

The PHA-based DPI under fasting/resting conditions was considered suitable for reference analysis, as it is reliably measurable, shows lower variability, and can be assessed under standardized conditions. The sample used consisted of *n* = 39 participants (no missing data), and the statistical distribution can be considered normal (*p* = 0.625, Shapiro–Wilk).

Exploratory analyses were performed to assess potential associations between the fasting/resting PHA-based DPI and demographic or anthropometric variables. No statistically significant associations were observed for age, sex, body mass index (BMI), or body surface area (BSA). Consequently, stratification of the proposed physiological reference threshold according to these variables was not considered justified within the present cohort.

The 90th percentile was therefore selected as a pragmatic and statistically more robust estimate of the upper physiological range. In contrast to conventional population-based reference intervals—such as those recommended by CLSI guidelines—which typically employ the 95th or 97.5th percentile, focusing on more extreme cutoffs in a sample of similar size would lack statistical stability. In this scenario, a 97.5th percentile would be determined largely by the highest individual observations within the sample and would therefore be highly sensitive to single outliers. A reliable application of formal CLSI-guided reference interval methodology is therefore inherently reserved for substantially larger cohorts. To quantify the uncertainty associated with the chosen threshold, bootstrap resampling was performed to derive confidence intervals for the upper reference limit. Based on this approach, the empirical 90th percentile was determined to be 0.3039 (≈ 0.30), indicating that 90% of healthy individuals had a DPI ≤ 0.30.

Bootstrap simulation with 10,000 samples yielded a 95% confidence interval of 0.29 to 0.34 for the upper limit. Within these boundaries, the true 90th percentile of the DPI in healthy individuals is expected to lie with 95% confidence.

**Figure 7:**
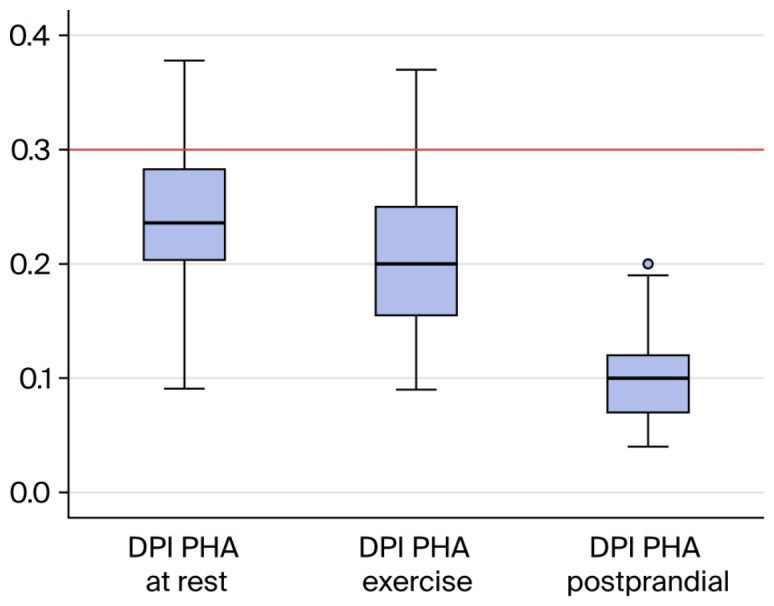
Upper physiological reference limit of the Doppler perfusion index.

Alternatively, calculation of the 90th percentile based on a normal distribution with a mean of 0.242 and a standard deviation of 0.057 resulted in a value of 0.315, which was close to the empirical estimate (Figure 7).

Note. Distribution of the proper hepatic artery-based Doppler perfusion index (DPI) under fasting/resting conditions. The horizontal line indicates the upper physiological reference limit (90th percentile; DPI = 0.30). Circles indicate outliers.

## 4. Discussion

The Doppler perfusion index has been available for more than two decades; however, despite its potential as a noninvasive functional parameter for assessing hepatic parenchymal disease, its methodological development has remained limited. Conceptually, the DPI was not intended to detect manifest structural lesions but rather to reflect hepatic involvement below the threshold of morphological imaging through alterations in hepatic hemodynamics, an approach explored particularly in the context of occult hepatic metastases (Leen, 1999; Macrì et al., 2012) [5,21].

Conceptually, the DPI shares certain similarities with modern molecular biomarkers such as circulating tumor DNA, not because both methods assess the same biological processes, but because they rely on a comparable diagnostic principle: the detection of indirect biological signals that may precede morphologically detectable disease. Whereas ctDNA reflects such signals at a molecular level (Tie et al., 2022) [22], the DPI reflects them at a functional hemodynamic level. However, the present study was not designed to evaluate such applications, and their clinical relevance remains to be established in prospective studies.

As a functional, derived parameter, its clinical applicability depends critically on a standardized and reproducible measurement approach. The literature on normative hepatic DPI is genuinely sparse, and the studies that do include healthy controls have produced inconsistent reference values—largely because they used different measurement protocols, fasting conditions, and operator techniques (Fowler et al., 1998; Oppo et al., 1998) [23,24]. The available literature is heterogeneous and largely limited to either portal venous flow analyses under varying physiological conditions (Ohnishi et al., 1985) [25] or arterial assessment using surrogate indices such as resistance or pulsatility indices (Öztürk et al., 2022; Paulson et al., 1996) [26,27]. While these limitations are partly attributed to methodological constraints of Doppler-based flow quantification (Ignee et al., 2016) [28]—particularly on the arterial side—this interpretation is not uniform and is contradicted by our previous work (Lueders et al., 2024) [20].

Studies combining volumetric measurements of portal venous and arterial inflow are scarce and largely limited to fasting conditions, small cohorts, and older Doppler technology; systematic intraindividual comparisons under defined physiological states remain uncommon. This limitation is particularly relevant for the Doppler perfusion index because it is derived from two separately measured flow variables and therefore represents a relational rather than a direct measurement. As described for ratio variables (Curran-Everett, 2013) [10] and confirmed by our own analyses (Lueders et al., 2024) [20], its behavior cannot be inferred from the individual flow parameters alone.

Consequently, the variations in the DPI across different physiological states reflect shifts in the balance between arterial and portal venous inflow, which are functionally coupled but governed by distinct regulatory mechanisms (Lautt, 2009) [2]. Our data demonstrate a concordant reduction in both arterial and portal venous blood flows during physical exercise, leaving the DPI relatively stable. In contrast, the postprandial state induces a discordant hemodynamic shift. Following food intake, portal venous influx increases, whereas arterial flow decreases. This opposing directional change in the volumetric flows results in a higher volatility of the resulting DPI quotient. The underlying arterial adaptation is better observable in the proper hepatic artery (PHA), which represents the definitive arterial influx to the liver parenchyma. This observation aligns with the classical theory of the hepatic arterial buffer response (HABR), which provides a plausible theoretical framework for this opposing regulatory dynamic. These distinct directional dynamics demonstrate why postprandial fasting is a prerequisite for reproducible clinical measurements, whereas a rest period prior to the examination is of minor clinical relevance for the DPI. The proposed upper reference threshold of 0.30 serves to delineate these normal physiological baseline variations from pathological increases in the hepatic arterial fraction, thereby providing a stable interpretative framework. Notably, the identified upper reference limit of 0.30 corresponds numerically to previously reported values (Leen et al., 2000) [7], despite substantial differences in the underlying methodological approach and derivation of the cutoff value.

Reflecting these physiological considerations, the behavior described above provides a direct rationale for selecting the arterial measurement site. Not only does the obvious presence of the non-hepatic CHA flow, which may be subject to different regulatory mechanisms, argue in favor of the PHA, but our measured data also show that the PHA exhibits lower variability. This difference in variability is at least partly explained by the fact that the volumetric flow calculation of the CHA is frequently compromised by unfavorable ultrasound insonation angles, leading to an increased mathematical vulnerability to measurement errors. The combination of these anatomical confounding factors and technical limitations may contribute to the explanation for the inconsistencies in earlier studies, which often failed to strictly differentiate between the two measurement sites. For a reproducible and liver-specific assessment of the DPI, the PHA should therefore preliminarily be preferred as the methodological standard. However, due to the numerous confounding factors and the inherent complexity of these vascular interactions, a conclusive evaluation is not exhaustively possible at this stage and must be reserved for a separate, dedicated discussion. While the cohort size provides a robust foundation for establishing this physiological and methodological framework, it inherently limits broader translational conclusions, meaning that the definitive clinical utility of the DPI in patient populations remains to be established in larger independent studies.

The need for standardization and reproducibility of DPI measurement was clearly articulated early in a high-level clinical context (Fong, 2000) [29], yet this central methodological challenge has remained largely unaddressed to date and is substantially advanced by the present findings.

### Limitations

The reference analysis is based on a small, selected cohort of 39 healthy adults without relevant comorbidities or regular medication use. Although the intraindividual study design allows robust comparisons across defined physiological states, transferability of the findings to more heterogeneous clinical populations is limited. In particular, cardiovascular comorbidities, liver disease, and hemodynamically active medications may influence hepatic perfusion and thereby affect the Doppler Perfusion Index.

The limited sample size further restricts the precision with which upper reference limits can be estimated. Consequently, the proposed upper physiological reference threshold of 0.30 should be regarded as preliminary and requires confirmation in larger independent cohorts. In particular, estimation of more extreme reference percentiles would require substantially larger study populations to achieve sufficient statistical stability and precision.

This was a single-center study in which all examinations were performed by a single experienced investigator. This ensured a high degree of methodological consistency and minimized operator-dependent variability within the study. However, the study design does not permit direct assessment of intra- or interobserver variability. It should be noted that reproducibility of the underlying Doppler-based flow measurements has previously been demonstrated in an independent study using the same methodological approach (Lueders et al., 2024) [20]. Nevertheless, the applicability of the proposed protocol across investigators with different levels of experience and across other institutions remains to be established and requires further validation in independent cohorts.

Beyond the study-specific limitations outlined above, it should be acknowledged that Doppler-based quantification of visceral blood flow and calculation of the Doppler Perfusion Index (DPI) are inherently subject to several methodological constraints. These include the angle dependency of Doppler velocity measurements, assumptions regarding vessel geometry and cross-sectional shape, technical challenges associated with small-caliber vessels, uncertainty in vessel diameter determination, anatomical vascular variants, and limited accessibility of certain arterial segments. The present study directly confirmed some of these limitations. Quantitative assessment of the common hepatic artery was frequently restricted by unfavorable insonation angles, resulting in increasing susceptibility to measurement error with larger angles due to the mathematical properties of Doppler angle correction. Likewise, the small caliber of the proper hepatic artery requires particularly precise diameter determination, as even minor measurement inaccuracies may disproportionately affect calculated volumetric flow because of the quadratic relationship between vessel diameter and cross-sectional area. Although these limitations are well recognized in visceral Doppler ultrasonography and cannot be discussed exhaustively within the scope of the present study, they remain important considerations when interpreting quantitative flow measurements and their potential clinical applications.

Despite improvements in methodological standardization and measurement consistency (Dietrich et al., 2001, 2007) [30,31], important biological and clinical questions regarding the DPI remain unresolved and warrant further investigation in future translational and clinical studies.

## 5. Conclusions

The Doppler perfusion index (DPI) is a physiologically dynamic ratio variable that reflects the immediate hemodynamic balance of the liver, serving as a foundational framework for its intended clinical applicability. This intraindividual study represents the first systematic evaluation of how different physiological states affect the parameter at the level of the calculated index.

Methodologically, the proper hepatic artery (PHA) should be preferred over the common hepatic artery (CHA) as the standard measurement site. The PHA provides superior liver-specific assessment and lower variability, as it avoids the angle-dependent Doppler-physical constraints and cosine-based correction errors associated with the frequently unfavorable anatomy of the CHA. By adhering to this site-specific selection within the framework of the established examination protocol, an empirical threshold of approximately 0.30 serves as a robust, preliminary upper physiological reference limit.

Ultimately, by successfully decoupling normal physiological baseline variations from pathological hemodynamics, this framework establishes a robust foundation for the translational clinical application of the Doppler perfusion index. Rather than presenting a distant perspective, the standardization and reference criteria developed in this study substantially advance the methodology toward its intended utility as a noninvasive functional marker capable of detecting early parenchymal or malignant changes before they become morphologically apparent.

## Figures and Tables

**Table 1:** Demographic and anthropometric characteristics of the healthy study population (*n* = 39).

Sex	Statistic	Age (Years)	Height (cm)	Weight (kg)	BSA (m^2^)	BMI (kg/m^2^)
Male (*n* = 21)	Mean	35.4	180.0	75.8	1.95	23.3
	SD	11.41	8.48	10.50	0.17	2.59
	Minimum	18	162	44	1.43	16.8
	Median	36	180	80	1.95	23.5
	Maximum	56	199	90	2.17	27.4
Female (*n* = 18)	Mean	40.4	167.1	61.1	1.68	21.9
	SD	8.81	5.56	10.55	0.13	3.90
	Minimum	24	156	45	1.47	17.37
	Median	40.5	166.5	60.0	1.65	21.88
	Maximum	58	176	95	2.02	34.90
Total (*n* = 39)	Mean	37.7	174.1	69.0	1.82	22.7
	SD	10.47	9.72	12.77	0.20	3.29
	Minimum	18	156	44	1.43	16.80
	Median	39	174	68	1.82	22.5
	Maximum	58	199	95	2.17	34.9

Note. BSA = body surface area calculated according to the DuBois formula; BMI = body mass index; SD = standard deviation.

**Table 2:** Descriptive statistics of the Doppler Perfusion Index (DPI) according to measurement site (CHA vs. PHA) and physiological condition (rest, post-exercise, postprandial) in healthy volunteers; sample size for each condition as indicated in row N.

Group	Statistic	DPI CHA at Rest	DPI CHA Post-Exertion	DPI CHA Postprandial	DPI PHA at Rest	DPI PHA Post-Exertion	DPI PHA Postprandial
TOTAL	N	25.0	22.0	26.0	39.0	39.0	38.0
	Mean	0.32	0.28	0.16	0.24	0.2	0.1
	SD	0.084	0.103	0.081	0.057	0.066	0.039
	Minimum	0.11	0.12	0.06	0.09	0.09	0.04
	Median	0.32	0.26	0.14	0.24	0.2	0.1
	Maximum	0.51	0.54	0.41	0.38	0.37	0.2

Note. CHA = common hepatic artery; PHA = proper hepatic artery; DPI = Doppler perfusion index. All values refer to repeated measurements within the same individuals under standardized physiological conditions.

## Data Availability

The data presented in this study are available on request from the corresponding author due to privacy and ethical restrictions.

## Supplementary Materials

The following supporting information can be downloaded at: https://www.mdpi.com/article/10.3390/diagnostics16121840/s1, Supplementary Table S1: Resting and exercise-related cardiovascular parameters; Supplementary Table S2: Exercise performance parameters; Supplementary Table S3: Vessel diameters under different physiological conditions; Supplementary Table S4: Resistive indices under different physiological conditions; Supplementary Table S5: Hepatic volumetric blood flow measurements under different physiological conditions; Supplementary Table S6: Doppler flow velocity measurements under different physiological conditions.

## Abbreviations

BMI	body mass index
BSA	body surface area
CHA	common hepatic artery
DPI	Doppler perfusion index
PHA	proper hepatic artery
PV	portal vein
RI	resistive index
SMA	superior mesenteric artery
