# Supplementary material for: Hepatic Doppler Perfusion Index in Healthy Adults: Standardization, Physiological Reference Limit, and Clinical Perspectives
Source: Diagnostics (Basel). 2026 Jun 14;16(12):1840. doi: 10.3390/diagnostics16121840 (PMC13298128; doi:10.3390/diagnostics16121840)
Supplement: Supplementary file 1 [file diagnostics-16-01840-s001.zip › Suppelemtary_TableS6_Vessel_Flow_Velocities.pdf]

Table S6. Doppler flow velocity measurements under different physiological conditions.

Time averaged flow velocities (Shift) of the common hepatic artery (CHA), proper hepatic artery (PHA), and portal vein (PV) at rest, during exercise, and postprandially in healthy volunteers (n = 39)

Sex	Statistic	CHA Rest (cm/s)	CHA Post-load (cm/s)	CHA Postprandial (cm/s)	PHA Rest (cm/s)	PHA Post-load (cm/s)	PHA Postprandial (cm/s)	PV Rest (cm/s)	PV Post-load (cm/s)	PV Postprandial (cm/s)
Male	Mean	24.0	18.4	20.1	26.5	20.2	19.4	12.2	10.8	18.5
	SD	5.36	5.81	6.70	5.13	5.70	5.14	2.17	2.09	2.92
	Minimum	16.1	10.4	11.9	15.4	11.1	11.4	8.1	6.4	14.6
	Median	23.55	18.40	18.70	27.30	20.30	19.15	12.10	10.50	18.00
	Maximum	31.4	28.0	34.7	34.6	33.3	31.8	15.2	16.2	27.1
Female	Mean	30.8	22.9	23.9	30.0	18.4	21.1	13.8	12.1	19.6
	SD	10.21	11.12	6.24	8.11	4.86	5.13	2.57	2.34	3.84
	Minimum	16.8	15.0	15.0	19.7	10.0	14.0	9.8	8.4	15.1
	Median	30.60	17.70	23.30	29.00	18.15	20.95	13.45	11.50	18.90
	Maximum	50.0	49.0	37.1	54.2	27.0	32.2	19.7	17.0	28.5

Table S6 (cont.)

Time averaged flow velocities (Shift) of the common hepatic artery (CHA), proper hepatic artery (PHA), and portal vein (PV) at rest, during exercise, and postprandially in healthy volunteers (n = 39)

Sex	Statistic	CHA Rest (cm/s)	CHA Post-load (cm/s)	CHA Postprandial (cm/s)	PHA Rest (cm/s)	PHA Post-load (cm/s)	PHA Postprandial (cm/s)	PV Rest (cm/s)	PV Post-load (cm/s)	PV Postprandial (cm/s)
**Total**	**Mean**	**27.0**	**20.7**	**22.0**	**28.1**	**19.4**	**20.2**	**13.0**	**11.4**	**19.0**
	**SD**	**8.40**	**8.96**	**6.63**	**6.81**	**5.34**	**5.13**	**2.47**	**2.27**	**3.38**
	**Minimum**	**16.1**	**10.4**	**11.9**	**15.4**	**10.0**	**11.4**	**8.1**	**6.4**	**14.6**
	**Median**	**26.40**	**18.05**	**22.55**	**27.30**	**18.30**	**19.95**	**13.20**	**11.00**	**18.50**
	**Maximum**	**50.0**	**49.0**	**37.1**	**54.2**	**33.3**	**32.2**	**19.7**	**17.0**	**28.5**

Note. CHA = common hepatic artery; PHA = proper hepatic artery; PV = portal vein; SD = standard deviation.
