# Supplementary material for: Hepatic Doppler Perfusion Index in Healthy Adults: Standardization, Physiological Reference Limit, and Clinical Perspectives
Source: Diagnostics (Basel). 2026 Jun 14;16(12):1840. doi: 10.3390/diagnostics16121840 (PMC13298128; doi:10.3390/diagnostics16121840)
Supplement: Supplementary file 1 [file diagnostics-16-01840-s001.zip › Supplementary TableS4_RI.pdf]

Supplementary Table S4. Resistive indices under different physiological conditions.

Resistive index (RI) of the common hepatic artery (CHA) and proper hepatic artery (PHA) at rest, post exertion, and postprandial, stratified by sex in healthy volunteers; sample size per condition given in row N.

Sex	Statistic	RI CHA at rest	RI CHA post-exertion	RI CHA postprandial	RI PHA at rest	RI PHA post-exertion	RI PHA postprandial
m	N	14.0	11.0	13.0	19.0	19.0	17.0
m	Mean	0.72	0.7	0.74	0.67	0.71	0.74
m	Std. Deviation	0.068	0.06	0.082	0.085	0.096	0.074
m	Minimum	0.59	0.59	0.59	0.53	0.54	0.61
m	Median	0.7	0.72	0.75	0.67	0.71	0.75
m	Maximum	0.84	0.8	0.87	0.9	0.95	0.87
w	N	11.0	11.0	13.0	18.0	18.0	18.0
w	Mean	0.71	0.73	0.76	0.67	0.68	0.74
w	Std. Deviation	0.05	0.064	0.068	0.084	0.105	0.115
w	Minimum	0.64	0.65	0.65	0.53	0.47	0.44

Sex	Statistic	RI CHA at rest	RI CHA post-exertion	RI CHA postprandial	RI PHA at rest	RI PHA post-exertion	RI PHA postprandial
w	Median	0.72	0.73	0.78	0.67	0.69	0.76
w	Maximum	0.8	0.89	0.87	0.9	0.95	0.87
**Total**	**N**	**25.0**	**22.0**	**26.0**	**37.0**	**37.0**	**35.0**
**Total**	**Mean**	**0.72**	**0.72**	**0.75**	**0.67**	**0.7**	**0.74**
**Total**	**Std. Deviation**	**0.059**	**0.062**	**0.075**	**0.083**	**0.1**	**0.096**
**Total**	**Minimum**	**0.59**	**0.59**	**0.59**	**0.53**	**0.47**	**0.44**
**Total**	**Median**	**0.71**	**0.72**	**0.78**	**0.67**	**0.71**	**0.75**
**Total**	**Maximum**	**0.84**	**0.89**	**0.87**	**0.91**	**0.95**	**0.87**

Note. CHA = common hepatic artery; PHA = proper hepatic artery; RI = resistive index.
