# Supplementary material for: Hepatic Doppler Perfusion Index in Healthy Adults: Standardization, Physiological Reference Limit, and Clinical Perspectives
Source: Diagnostics (Basel). 2026 Jun 14;16(12):1840. doi: 10.3390/diagnostics16121840 (PMC13298128; doi:10.3390/diagnostics16121840)
Supplement: Supplementary file 1 [file diagnostics-16-01840-s001.zip › Supplementary_TableS1_Heart_Rate_Parameters.pdf]

Supplementary Table S1. Resting and exercise-related cardiovascular parameters

Heart rate parameters at rest and during exercise in the healthy study population (n = 39)

Sex	Statistic	Resting HR (bpm)	Exercise HR (bpm)	Max HR (sex-specific)	Recalculated target HR
Male (n = 21)	Mean	68.8	159.7	188.4	152.5
	SD	12.61	10.86	7.30	6.56
	Minimum	45	137	175.2	140.6
	Median	70	161	188.0	151.8
	Maximum	91	176	199.5	162.1
Female (n = 18)	Mean	74.3	165.4	182.9	150.4
	SD	10.61	8.75	5.90	4.55
	Minimum	60	147	171.1	143.5
	Median	74	165	182.9	150.4
	Maximum	100	183	193.9	160.1
Total (n = 39)	Mean	71.4	162.3	185.9	151.5
	SD	11.91	10.23	7.15	5.75
	Minimum	45	137	171.1	140.6
	Median	72	164	185.4	150.9
	Maximum	100	183	199.5	162.1

Note. HR = heart rate; SD = standard deviation.
