# Supplementary material for: Hepatic Doppler Perfusion Index in Healthy Adults: Standardization, Physiological Reference Limit, and Clinical Perspectives
Source: Diagnostics (Basel). 2026 Jun 14;16(12):1840. doi: 10.3390/diagnostics16121840 (PMC13298128; doi:10.3390/diagnostics16121840)
Supplement: Supplementary file 1 [file diagnostics-16-01840-s001.zip › Supplementary_TableS2_Blood_Pressure_Workload.pdf]

Supplementary Table S2. Exercise performance parameters.

Blood pressure parameters at rest and during exercise and workload characteristics in the healthy study population (n = 39)

Sex	Statistic	SBP Rest (mmHg)	DBP Rest (mmHg)	SBP Exercise (mmHg)	DBP Exercise (mmHg)	Workload (W)	Workload (W/kg)
Male (n = 21)	Mean	113.1	79.5	163.1	76.1	175.2	2.31
	SD	9.99	8.53	23.72	15.68	43.40	0.51
	Minimum	94	62	130	51	75	1.33
	Median	113	79	160	80	175	2.35
	Maximum	134	104	214	100	250	3.57
Female (n = 18)	Mean	107.1	75.9	148.8	75.1	104.4	1.73
	SD	12.08	7.71	10.58	11.58	24.06	0.39
	Minimum	85	61	131	54	75	1.10
	Median	110	78	149.5	75	100	1.77
	Maximum	128	88	172	90	145	2.55

Supplementary Table S2 (cont.)

Blood pressure parameters at rest and during exercise and workload characteristics in the healthy study population (n = 39)

Sex	Statistic	SBP Rest (mmHg)	DBP Rest (mmHg)	SBP Exercise (mmHg)	DBP Exercise (mmHg)	Workload (W)	Workload (W/kg)
Total (n = 39)	Mean	110.3	77.8	156.5	75.6	142.6	2.04
	SD	11.27	8.26	19.96	13.77	50.29	0.54
	Minimum	85	61	130	51	75	1.10
	Median	110	79	152	77	140	1.92
	Maximum	134	104	214	100	250	3.57

Note. SBP = systolic blood pressure; DBP = diastolic blood pressure; SD = standard deviation.
