# Supplementary material for: Hepatic Doppler Perfusion Index in Healthy Adults: Standardization, Physiological Reference Limit, and Clinical Perspectives
Source: Diagnostics (Basel). 2026 Jun 14;16(12):1840. doi: 10.3390/diagnostics16121840 (PMC13298128; doi:10.3390/diagnostics16121840)
Supplement: Supplementary file 1 [file diagnostics-16-01840-s001.zip › Supplementary_TableS3_Vessel_Diameters.pdf]

Supplementary Table S3. Vessel diameters under different physiological conditions.

Diameter of the common hepatic artery (CHA), proper hepatic artery (PHA), and portal vein (PV) at rest, during exercise, and postprandially in healthy volunteers (n = 39)

Sex	Statistic	CHA Rest (mm)	CHA Post-load (mm)	CHA Postprandial (mm)	PHA Rest (mm)	PHA Post-load (mm)	PHA Postprandial (mm)	PV Rest (mm)	PV Post-load (mm)	PV Postprandial (mm)
Male (n = 20–21)	Mean	5.3	4.6	5.2	4.1	3.7	3.9	10.5	9.8	11.9
	SD	0.89	0.62	0.96	0.36	0.45	0.44	1.20	0.86	0.81
	Minimum	3.8	3.2	3.2	3.3	2.3	2.8	8.3	8.4	9.8
	Median	5.2	4.8	5.1	4.0	3.7	3.9	10.5	9.6	12.0
	Maximum	7.1	5.8	7.6	5.0	4.6	4.7	12.3	11.8	13.8
Female (n = 18)	Mean	4.9	4.4	4.6	3.8	3.4	3.6	10.1	9.0	11.8
	SD	0.57	0.52	0.67	0.22	0.44	0.28	1.26	1.24	1.32

Supplementary Table S3 (cont.)

Diameter of the common hepatic artery (CHA), proper hepatic artery (PHA), and portal vein (PV) at rest, during exercise, and postprandially in healthy volunteers (n = 39)

Sex	Statistic	CHA Rest (mm)	CHA Post-load (mm)	CHA Postprandial (mm)	PHA Rest (mm)	PHA Post-load (mm)	PHA Postprandial (mm)	PV Rest (mm)	PV Post-load (mm)	PV Postprandial (mm)
	Minimum	4.1	3.6	3.9	3.2	2.4	3.2	8.7	6.8	9.6
	Median	5.0	4.3	4.4	3.9	3.4	3.5	10.0	8.9	11.8
	Maximum	5.9	5.6	6.7	4.0	4.1	4.1	13.7	12.6	14.7
Total (n = 35–39)	Mean	5.2	4.5	4.9	3.9	3.6	3.8	10.3	9.4	11.9
	SD	0.77	0.58	0.86	0.35	0.47	0.40	1.23	1.11	1.06
	Minimum	3.8	3.2	3.2	3.2	2.3	2.8	8.3	6.8	9.6
	Median	5.1	4.5	4.9	3.9	3.6	3.8	10.4	9.3	12.0
	Maximum	7.1	5.8	7.6	5.0	4.6	4.7	13.7	12.6	14.7

Note. CHA = common hepatic artery; PHA = proper hepatic artery; PV = portal vein; SD = standard deviation.
