# Supplementary material for: Hepatic Doppler Perfusion Index in Healthy Adults: Standardization, Physiological Reference Limit, and Clinical Perspectives
Source: Diagnostics (Basel). 2026 Jun 14;16(12):1840. doi: 10.3390/diagnostics16121840 (PMC13298128; doi:10.3390/diagnostics16121840)
Supplement: Supplementary file 1 [file diagnostics-16-01840-s001.zip › Supplementary_TableS5_Flow_Rates.pdf]

Supplementary Table S5. Hepatic volumetric blood flow measurements under different physiological conditions.

Flow rates (ml/min) in the common hepatic artery (CHA), proper hepatic artery (PHA), and portal vein (PV) at rest, post-load, and postprandial, stratified by sex in healthy volunteers; sample size per condition given in row N.

Sex	Statistic	Flow CHA at rest [ml/min]	Flow CHA post-load [ml/min]	Flow CHA postprandial [ml/min]	Flow PHA at rest [ml/min]	Flow PHA post-load [ml/min]	Flow PHA postprandial [ml/min]	Flow PV at rest [ml/min]	Flow PV post-load [ml/min]	Flow PV postprandial [ml/min]
m	N	14.0	11.0	13.0	21.0	21.0	20.0	21.0	21.0	21.0
m	Mean	286.7	181.6	251.0	210.6	132.8	144.5	638.8	487.1	1237.0
m	Std. Deviation	85.44	68.92	132.93	49.45	46.71	52.91	161.95	111.35	227.95
m	Minimum	109.5	62.7	82.9	89.7	50.8	54.6	350.4	244.2	954.4
m	Median	309.15	183.8	245.0	222.0	136.5	139.15	627.4	475.0	1221.6
m	Maximum	394.8	291.3	549.8	277.5	250.9	264.2	1033.2	659.9	1931.0
w	N	11.0	11.0	13.0	18.0	18.0	18.0	18.0	18.0	18.0
w	Mean	332.6	224.1	248.7	199.8	102.7	130.8	671.3	472.5	1289.2
w	Std. Deviation	128.4	184.63	168.29	58.6	42.76	46.9	194.93	167.81	293.55

Supplementary Table S5 (cont.)

Flow rates (ml/min) in the common hepatic artery (CHA), proper hepatic artery (PHA), and portal vein (PV) at rest, post-load, and postprandial, stratified by sex in healthy volunteers; sample size per condition given in row N.

Sex	Statistic	Flow CHA at rest [ml/min]	Flow CHA post-load [ml/min]	Flow CHA postprandial [ml/min]	Flow PHA at rest [ml/min]	Flow PHA post-load [ml/min]	Flow PHA postprandial [ml/min]	Flow PV at rest [ml/min]	Flow PV post-load [ml/min]	Flow PV postprandial [ml/min]
w	Minimum	167.4	107.4	118.7	141.1	38.2	80.5	427.7	235.9	716.2
w	Median	327.9	142.8	206.1	181.35	98.45	122.75	616.05	447.4	1352.6
w	Maximum	565.4	723.7	784.4	388.2	213.7	254.9	1211.1	882.4	1744.5
**Total**	**N**	**25.0**	**22.0**	**26.0**	**39.0**	**39.0**	**38.0**	**39.0**	**39.0**	**39.0**
**Total**	**Mean**	**306.9**	**202.9**	**249.9**	**205.6**	**118.9**	**138.0**	**653.8**	**480.4**	**1261.1**
**Total**	**Std. Deviation**	**106.61**	**137.72**	**148.58**	**53.41**	**46.88**	**49.97**	**176.28**	**138.48**	**258.06**
**Total**	**Minimum**	**109.5**	**62.7**	**82.9**	**89.7**	**38.2**	**54.6**	**350.4**	**235.9**	**716.2**
**Total**	**Median**	**312.1**	**158.8**	**207.85**	**205.7**	**108.8**	**128.75**	**627.4**	**470.8**	**1244.7**

Sex	Statistic	Flow CHA at rest [ml/min]	Flow CHA post-load [ml/min]	Flow CHA postprandial [ml/min]	Flow PHA at rest [ml/min]	Flow PHA post-load [ml/min]	Flow PHA postprandial [ml/min]	Flow PV at rest [ml/min]	Flow PV post-load [ml/min]	Flow PV postprandial [ml/min]
w	Minimum	167.4	107.4	118.7	141.1	38.2	80.5	427.7	235.9	716.2
**Total**	**Maximum**	**565.4**	**723.7**	**784.4**	**388.2**	**250.9**	**264.2**	**1211.1**	**882.4**	**1931.0**

Note. CHA = common hepatic artery; PHA = proper hepatic artery; PV = portal vein. Flow values represent volumetric flow rates (ml/min) derived from Doppler ultrasound measurements under standardized conditions (rest, post-load, postprandial).
